# Hepatitis E virus infects human testicular tissue and Sertoli cells

**DOI:** 10.1080/22221751.2024.2332657

**Published:** 2024-03-22

**Authors:** Tianxu Liu, Yalei Cao, Jiaming Weng, Songzhan Gao, Zirun Jin, Yun Zhang, Yuzhuo Yang, He Zhang, Changyou Xia, Xin Yin, Yong Luo, Qiyu He, Hui Jiang, Lin Wang, Zhe Zhang

**Affiliations:** a Department of Microbiology and Infectious Disease Center, School of Basic Medical Sciences, Peking University Health Science Center, Beijing, People’s Republic of China; b Department of Urology, Peking University Third Hospital, Beijing, People’s Republic of China; c Center for Reproductive Medicine, Peking University Third Hospital, Beijing, People’s Republic of China; d Department of Andrology, Third Affiliated Hospital of Zhengzhou University, Zhengzhou, People’s Republic of China; e Department of Urology, Peking University First Hospital, Beijing, People’s Republic of China; f State Key Laboratory for Animal Disease Control and Prevention, Harbin Veterinary Research Institute, Chinese Academy of Agricultural Sciences, Harbin, People’s Republic of China

**Keywords:** Hepatitis E virus; chronic hepatitis E; extrahepatic replication; human testis; Sertoli cells

## Abstract

Globally, hepatitis E virus (HEV) infections are prevalent. The finding of high viral loads and persistent viral shedding in ejaculate suggests that HEV replicates within the human male genital tract, but its target organ is unknown and appropriate models are lacking. We aimed to determine the HEV tropism in the human testis and its potential influence on male reproductive health. We conducted an ex vivo culture of human testis explants and in vitro culture of primary human Sertoli cells. Clinically derived HEV genotype 1 (HEV1) and HEV3 virions, as well as rat-derived HEV-C1, were used for inoculation. Transcriptomic analysis was performed on testis tissues collected from tacrolimus-treated rabbits with chronic HEV3 infection. Our findings reveal that HEV3, but not HEV1 or HEV-C1, can replicate in human testis explants and primary human Sertoli cells. Tacrolimus treatment significantly enhanced the replication efficiency of HEV3 in testis explants and enabled successful HEV1 infection in Sertoli cells. HEV3 infection disrupted the secretion of several soluble factors and altered the cytokine microenvironment within primary human Sertoli cells. Finally, intratesticular transcriptomic analysis of immunocompromised rabbits with chronic HEV infection indicated downregulation of genes associated with spermatogenesis. HEV can infect the human testicular tissues and Sertoli cells, with increased replication efficiency when exposed to tacrolimus treatment. These findings shed light on how HEV may persist in the ejaculate of patients with chronic hepatitis E and provide valuable ex vivo tools for studying countermeasures.

## Introduction

Hepatitis E, an infectious disease caused by hepatitis E virus (HEV) infection is currently the major acute viral hepatitis problem worldwide. HEV is a single-stranded positive-sense RNA virus that mainly transmits via fecal-oral route. HEV genotype 1 to 4 (HEV1-4) are the four main genotypes that induce most human hepatitis E cases [1]. Recently, emerging reports have found that HEV stains of species *Rocahepevirus* from rats can also infect humans [2, 3]. Most HEV infections are acute and typically self-limiting. However, a minority of patients with hepatitis E develop liver failure, particularly those in pregnancy and with underlying chronic liver disease [4]. Immunocompromised individuals, such as organ transplant recipients are particularly vulnerable to chronic HEV infection [1]. Moreover, although HEV predominantly targets the liver, there is compelling evidence suggesting a causal effect causal effect between HEV infection and extrahepatic manifestations [5].

During chronic HEV infection, HEV replicates more efficiently under immunosuppressant treatment and extrahepatic replication and manifestations can frequently occur [4, 5]. HEV replication outside of the liver and hepatocytes has been reported in cell culture models, including neuronal [6], renal [7] and placental cell lines [8], as well as in tissues of hepatitis E patients or experimentally infected animals such as kidney [9], brain [10], cerebrospinal fluid [11], urine [12], placenta [13] and intestine [14]. In recent years, the presence of HEV in human semen samples has aroused broad interest in the field. It was first reported in 2018 by a Chinese group that a high detection rate of 28% for HEV4 sequences in the semen of infertile men was found [15]. However, confounding results were subsequently reported by ours [16] and groups in Germany [17] and Egypt [18] with no detection of HEV RNA in semen of infertile males. The potential association between HEV infection and male infertility remains a subject for future investigation.

The implications of detecting HEV RNA in infertile males and chronic HEV-infected patients hold distinct and separate scientific significance. HEV resembles characteristics similar to many other zoonotic RNA viruses, such as SARS-CoV-2, Zika Virus and Ebola virus. One of the intriguing characteristics of the abovementioned viruses is that they have broad tissue tropism [19–21]. The male genital system has been well-characterized as a favorable niche for Zika virus and Ebola virus [22, 23]. Recently, a German group reported that HEV can be persistently detected (>9 months) in the ejaculates of most chronic hepatitis E patients (75%, 9/12)[24, 25]. HEV RNA in the ejaculate can be significantly higher than in serum or plasma and stool. Moreover, Enveloped HEV virions were seen in the ejaculate using immunogold electron microscopy. This group subsequently proved that HEV in a patient’s ejaculate can infect PLC/PRF/5 cells indicating the shedding of infectious HEV virions [25]. Such evidence was observed in chronic hepatitis E patients, strongly suggesting tropism of HEV for the testis. However, direct evidence of HEV infection within the human testis is still pending, and further investigation is needed to unravel the potential interplay between the virus and the host within such tissues.

In this study, through the infection of human testicular tissue explants and primary human Sertoli cells with HEV1 and HEV3, we demonstrated that HEV genotypes are capable of replicating within human testicular cells and generating infectious viral particles. Furthermore, we delved into the transcriptional landscape associated with HEV infection in the testicular tissue of an immunocompromised rabbit model, which mimics chronic hepatitis E patients.

## Materials and methods

### Ethics approval

Adult human testicular samples were obtained from patients with obstructive azoospermia undergoing surgical testicular sperm extraction for in vitro fertilization. Informed consent was obtained from all patients prior to surgery. This study was approved by the ethics committee of Peking University Third Hospital (IRB00006761-M2022692) and complied with the criteria stated by the Declaration of Helsinki.

### Virus strains

The rabbit-derived HEV-3ra, human-derived HEV1 (subtype 1b) and HEV3 (subtype 3b), and rat-derived HEV-C1 (kindly provided by Dr. Tiancheng Li) strains were all made from feces (GenBank accession No. JX109834, JQ655734, MF996356 and JX120573). To prepare these strains for the experiment, the HEV-positive fecal samples were diluted in sterile phosphate buffer saline respectively to make 10∼20% (wt/vol) suspensions and then centrifuged at 5,000×g at 4 °C for 20 minutes. The clarified suspension was filtered through 0.45 and 0.22 μm filters and titrated subsequently.

### Organotypic culture of human testicular tissues and infection

Human testicular tissues were cultured as described previously [26, 27]. Briefly, once the human testicular sample was obtained, it was washed three times using PBS immediately and dissected into approximately 2-mm^3^ fragments using sterile scissors. These tissue fragments were cultured in a 35-mm dish containing DMEM/F12 medium supplemented with 1 × nonessential amino acid, 1×ITS (human insulin, human transferrin, and sodium selenite), 1% penicillin and streptomycin, and 10% fetal bovine serum. The human testicular tissues were incubated with approximately 2 × 10^7^ copies of HEV for 24 hours at 35°C, 5% CO_2_. Then virus was removed by washing with 1×PBS for five times and fresh medium was added. Supernatant at 0 dpi was collected after washing before culture. The residual value obtained for day 0 was subtracted from each kinetic point. The supernatants from cultured human testicular tissues were harvested, clarified through centrifugation, and subsequently stored at -80°C. The testicular tissues were fixed in 4% paraformaldehyde for immunofluorescence staining or stored at −80°C for RNA extraction.

Additional methods and materials are provided in Supplementary Materials.

## Results

### HEV3 replicates in human testicular tissue and releases infectious virions

**Figure 1.**
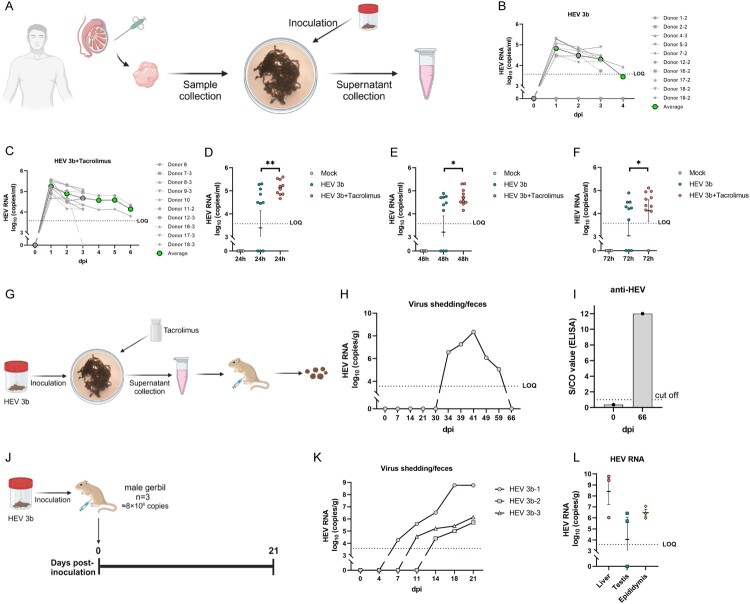
HEV replication in human testicular tissues. (A) Experimental design. Testis tissues were collected from male donors and cultured as tissue explants for ex vivo infection of HEV. Supernatants were collected for HEV RNA detection. (B) HEV RNA in supernatants of human testis explants inoculated with HEV genotype 3b. (C) HEV RNA in supernatants of human testis explants inoculated with HEV genotype 3b and treated with tacrolimus. (D) Comparison of HEV RNA levels in the supernatants collected from testis explants inoculated with HEV-3b and HEV-3b treated with tacrolimus at 24 hours post-inoculation (hpi), (E) 48 hpi and (F) 72 hpi. (G-I) Supernatants collected from testis explants inoculated with HEV-3b and treated with tacrolimus were inoculated to a Mongolian gerbil. (G) Experimental design. (H) The level of fecal HEV shedding of this gerbil was monitored. (I) Detection of anti-HEV antibodies in gerbil’s serum samples at 66 dpi. (J-L) HEV-3b infection experiment in gerbils. (J) Experimental design. (K) The levels of fecal HEV shedding of gerbils were monitored. (L) HEV RNA levels in liver, testis and epididymis tissues collected at 21 dpi. dpi, day post-inoculation; HEV, hepatitis E virus; hpi, hour post-inoculation; LOQ, lower limit of quantification; Tac, tacrolimus.

Testis explants were obtained from 19 uninfected donors (some tissue from the same donor was separated into 2–3 independent explants). Human testicular tissues were cultured as described previously [26, 27]. The observed proliferating cell nuclear antigen (PCNA) signals signify a high potential for proliferation and mitotic activity in the cultured tissues (Supplementary Figure 1). These explants were cultured and exposed to HEV3, HEV1, and HEV-C1 (rat HEV) derived from fecal samples ex vivo, following previously established protocols (Figure 1(A)). HEV3 can replicate in 70.0% (7/10) of the donors’ testis explants, resulting in the release of HEV RNA into the culture supernatant. RT-qPCR analysis revealed a substantial replication of HEV3 that reached a plateau within 2 days in these testes (Figure 1(B)).

Previous studies demonstrated that seminal shedding of HEV occurred in HEV3-infected chronic hepatitis E patients treated with immunosuppressants but not acute hepatitis E patients [24]. Therefore, we applied tacrolimus to mimic the immunosuppressed environment. To better understand the effect of tacrolimus on HEV, 10 donors’ tissues were divided into several explants, with one of them receiving tacrolimus treatment. Indeed, treatment of tacrolimus increased the susceptibility of testis explants to HEV as all treated explants (100.0%, 10/10) showed HEV replication compared to 70% (7/10) in the untreated group (Figure 1(C)). At 24, 48 and 72 hours post-inoculation (hpi), tacrolimus-treated explants showed a significantly higher HEV RNA level in the supernatants than the untreated explants (Figure 1(D, E and F)). We also inoculated the testis explant with HEV1 and HEV-C1 and neither genotype showed signs of replication even when treated with tacrolimus (Supplementary Figure 2).

Culturing wild-type HEV strains in cells has proven to be challenging. To assess the capability of testes to generate infectious HEV particles, we conducted experiments using gerbils, small rodents susceptible to human HEV-3. A female gerbil was intraperitoneally inoculated with the pooled supernatants collected from infected testis explants at 1 dpi (Figure 1(G)). Subsequently, stable fecal virus shedding was observed starting from 34 dpi, demonstrating the infectivity of viral progeny (Figure 1(H)). Anti-HEV antibodies were detected at 66 dpi (Figure 1(I)). We intraperitoneally inoculated three male gerbils (≈8×10^6^ copies/gerbil) with the same HEV-3b strain that we used to inoculate the human testicular tissue explants (Figure 1(J)). HEV infection was induced as fecal virus shedding started from 7 to 14 dpi (Figure 1(K)). All gerbils were euthanized at 21 dpi and liver, testis and epididymis tissues were collected for HEV RNA detection. HEV RNA was detected in all gerbils liver and epididymis tissues, and two out of three gerbils testis were also tested positive (Figure 1(L)).

Altogether, these data collectively illustrate that HEV3 could infect and replicate in the human testis ex vivo, leading to the production of infectious viral particles. Moreover, the introduction of tacrolimus treatment significantly enhanced the efficiency of viral replication in the testis explants.

### HEV3 infects sertoli cells and germ cells in human testis explants

**Figure 2.**
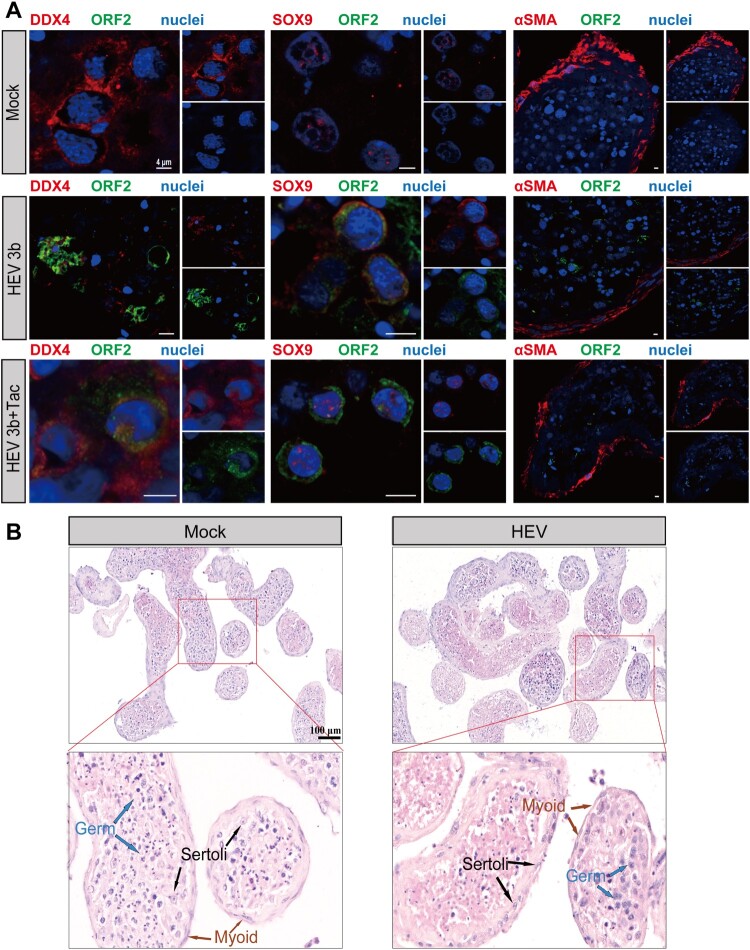
Characterization and morphology of HEV-infected human testicular cells ex vivo. (A) Immunofluorescence analysis for HEV ORF2 protein and cell markers identified HEV ORF2 protein in DDX4^+^ germ cells and SOX9^+^ Sertoli cells, especially in tacrolimus-treated testis tissues infected with HEV genotype 3b (white arrows). No obvious staining was seen in α-SMA^+^ peritubular cells. Staining for HEV ORF2 was not observed in Mock group testis. Nuclei are stained in blue. Scale bars: 20 μm. (B) Representative field of view of HE stained sections prepared from the testis tissues inoculated with HEV and Mock group. Scale bars: 100 μm. HEV, hepatitis E virus; Tac, tacrolimus.

We conducted immunofluorescence analysis on testis explants that were either mock-infected or infected with HEV. Antibodies against human testicular cell markers, including DEAD-Box helicase 4 (DDX4) for germ cells, SRY-box9 (SOX9) for Sertoli cells and α-smooth muscle actin (α-SMA) for testicular peritubular-myoid cells were used. Compared with no positive signal in the mock-infected negative group, strong ORF2 staining for HEV-3b was observed within the majority of seminiferous tubules. Within these tubules, spotty fluorescent HEV-3b ORF2 staining was found to co-localize with DDX4-positive testicular cells, indicating the infection of germ cells. Furthermore, SOX9-positive Sertoli cells also exhibited co-staining with HEV-3b ORF2. And, a weaker spotty staining pattern was observed within the seminiferous tubule wall, where colocalized α-SMA-positive myoid peritubular cells were presented (Figure 2(A)).

Taken together, these findings provide strong evidence that HEV exhibits a tropism for both germ cells and somatic cells within the human testis.

### HEV3 infection is associated with tissue injury

To date, whether HEV can induce injury in human testis tissues remains unknown, as no testis biopsy samples were collected from HEV-infected patients for examination. Accordingly, we assessed whether HEV3 infection impairs the morphology of the human testis explants. Hematoxylin-Eosin (HE) staining demonstrates that, at 3 dpi, mock-infected controls exhibited well-organized germ cells, with some sperm visible within the seminiferous tubules. In contrast, HEV3-infected testis explants displayed a thinning of the seminiferous epithelium, disordered arrangement, and a loss of germ cells within the seminiferous tubules (Figure 2(B)). Our findings indicate that HEV3 infection induces elevated damage to testis tissue explants, impacting the quantity and organized distribution of germ cells.

We then evaluated the impact of HEV3 infection on the testicular secretome by quantifying the concentrations of soluble factors in the supernatants. While HEV3 did induce a modest increase in certain pro-inflammatory cytokines, these changes were not statistically significant. It is noteworthy that the pro-inflammatory factor IL-18 exhibited a significant increase at 72 hours post-inoculation (Supplementary Figure 3).

### HEV1 and HEV3 infect primary human Sertoli cells and HEV3 alters the cells’ secretome

**Figure 3.**
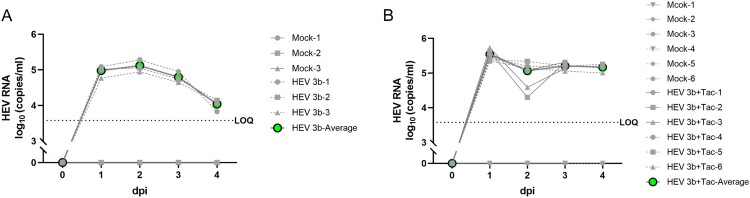
HEV replication in human primary Sertoli cells. HEV-3b was used to inoculate the primary human Sertoli cells. HEV RNA in the supernatants was quantified by RT-qPCR over time. HEV RNA in supernatants of human primary Sertoli cells infected with (A) HEV-3b or (B) additionally treated with tacrolimus. Prolonged release of HEV RNA in supernatants was seen in tacrolimus-treated Sertoli cells. dpi, day post-inoculation; HEV, hepatitis E virus; LOQ, lower limit of quantification; Tac, tacrolimus.

Based on the results of human testis explants, positive staining of HEV ORF2 can be observed in Sertoli cells. To further explore the potential productive infection of Sertoli cells, we cultured and inoculated primary human Sertoli cells with HEV1 and HEV3 strains and monitored viral replication dynamics over time. RT-qPCR analysis of supernatants demonstrated that HEV3 can indeed infect primary human Sertoli cells. The HEV3 replication reached a peak at about 2 dpi and the HEV RNA levels started to decline thereafter (Figure 3(A)). Treatment of tacrolimus prolonged the infection as no obvious decline of HEV RNA levels after 2 dpi (Figure 3(B)). In contrast, the efficiency of HEV1 infection in human Sertoli cells was lower compared to HEV3. Successful HEV1 infection can be established only when tacrolimus is added to the experimental setup (Supplementary Figures 4A and 4B).

**Figure 4.**
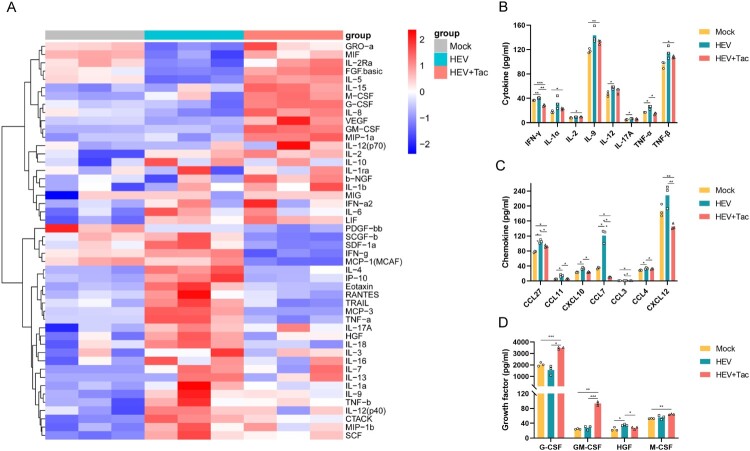
Primary human Sertoli cells secretome after HEV infection. Supernatants’ soluble factors determined by Human 48-Plex Luminex assay in Sertoli cells infected with HEV-3b with (*n* = 3) or without (*n* = 3) tacrolimus treatment, and Mock group (n = 3) at 3 dpi. (A) The color code of the heatmap represents a fold change in the level of the soluble factors in supernatants between HEV-infected, HEV-infected with tacrolimus treatment and time-matched mock-infected controls. The (B) cytokines, (C) chemokines and (D) growth factors in the supernatant from each group. *p < 0.05, ** *p* < 0.01, *** *p* < 0.001 and ****p < 0.0001 values are for HEV-infected, HEV-infected with tacrolimus treatment and time-matched mock-infected controls.

As HEV3 replicated more efficiently than HEV1 in Sertoli cells, we next investigated whether HEV3 infection impairs the dynamic secretory function of primary human Sertoli cells. Three days post-infection, supernatants were collected from HEV3, HEV3-Tacrolimus, or mock-infected Sertoli cell cultures, and soluble factors were subsequently quantiﬁed. Compared to the mock group, HEV3 infection without tacrolimus treatment resulted in a signiﬁcant increase of several pro-inﬂammatory mediators, including TNF-α, IL-2, IL-6, sICAM-1, CCL-3, CCL-4, G-CSF, and GM-CSF in human Sertoli cells (Figure 4(A and B)).

### Transcriptome analysis of intratesticular host response to chronic HEV infection in rabbits

**Figure 5.**
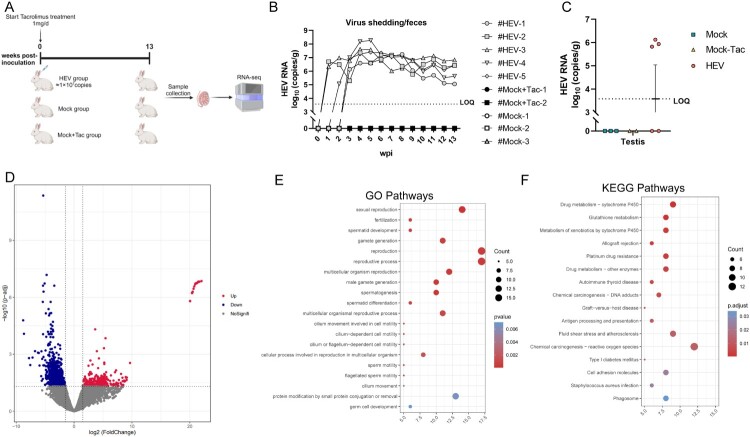
Intratesticular transcriptome analysis of tacrolimus-treated rabbits with chronic HEV3 infection. (A) Experimental scheme of the establishment of chronic HEV3 infection in rabbits. Rabbits in HEV and Mock + Tac groups were administrated orally with tacrolimus (1 mg/d per rabbit) starting from inoculation. (B) Quantification of HEV RNA by RT-qPCR in fecal samples of rabbits in each group. (C) Quantification of HEV RNA by RT-qPCR in testicular tissues collected from rabbits in each group at 13 weeks post-inoculation. (D) Comparison of gene expressions in HEV RNA-positive testis tissues (*n* = 3) from the chronic HEV3 infection group versus testis tissues (*n* = 2) from Mock + Tac group by volcano plot. Benjamini-Hochberg (BH)-corrected *p*-value (*p* adjust) < 0.05 and |log2 Fold Change| > 1. (E) Dot plot visualization of enriched GO terms in significant down-regulation genes. The color of the dots represents the BH-corrected *p*-value (*p* adjust) for each enriched Gene Ontology (GO) term, and the size represents the number of enriched genes. The rich factor represents the ratio of the number of genes enriched in the GO term to the annotation genes in the background. *p* adjust < 0.05 was considered significant. Pathways are shown and ordered according to significance. (F) KEGG enrichment pathway analysis of upregulated DEGs. Significantly enriched pathways in HEV RNA-positive testis tissues (*n* = 3) from the chronic HEV3 infection group versus testis tissues (*n* = 2) from the Mock + Tac group. *p* adjust < 0.05 was considered significant. Pathways are ordered according to significance.

The intratesticular host response to chronic HEV infection is still unknown. Given the difficulty in acquiring suitable human samples, we used rabbits with chronic HEV infection as a surrogate (Figure 5(A)). Tacrolimus was administrated daily via gavage for seven consecutive days with 1 mg/d once daily after HEV inoculation and reduced to every two days from the second week. Five rabbits were inoculated with HEV3 and received tacrolimus treatment (HEV group). Another five rabbits were inoculated with HEV-negative inocula, and of them, two rabbits received tacrolimus treatment (Mock + Tac) and three did not (Mock group). We have established chronic HEV3 (rabbit HEV) infection in tacrolimus-treated rabbits characterized by persistent fecal HEV shedding for about 13 weeks (Figure 5(B)). At 13 weeks post-inoculation, rabbits were euthanized and three male rabbits with positive HEV RNA in the testis tissues were selected for the present study (Figure 5(C)). Transcriptome analysis was performed with testis tissues collected from three chronically infected rabbits and two tacrolimus-treated mock-infected rabbits. Compared with mock-infected rabbits, 1208 differentially expressed genes (DEGs) were identified in the chronically infected group (Supplementary Figure 5). Among these DEGs, 887 were down-regulated, including genes associated with spermatid differentiation and development, such as testis-specific serine kinase 3 (*Tssk3*), polypeptide N-acetylgalactosaminyltransferase like 5 (*Galntl5*), outer dense fiber of sperm tails 1 (*Odf1*), zona pellucida glycoprotein 1 (*Zp1*), hook microtubule-tethering protein 1 (*Hook1*) and serine protease 37 (*Prss37*). These genes, like Galntl5, Hook1, and Prss37, are crucial for spermatid differentiation, sperm migration, and hyperactivation (Figure 5(D)). Gene Ontology (GO) enrichment analysis of these down-regulated genes enriched in spermatid differentiation, spermatid development and sperm motility (Figure 5(E)). Kyoto Encyclopedia of Genes and Genomes (KEGG) enrichment analysis showed that the up-regulated DEGs were significantly enriched in pathways related to graft-versus-host disease and chemical carcinogenesis-reactive oxygen species (Figure 5(F)).

Subsequently, we investigated whether long-term tacrolimus treatment would affect the reproductive health of rabbits in our experiment. Compared with Mock rabbits, only 4 DEGs were identified in the Mock + tacrolimus rabbits (Supplementary Figure 6A). Unsupervised principal component analysis (PCA) showed no obvious changes between the Mock and Mock + Tacrolimus groups (Supplementary Figure 6B). These results support the conclusion that 13-week tacrolimus treatment on rabbits exerts a limited influence on the intratesticular transcriptome.

Mild injury within the seminiferous tubules was observed in one out of three chronically infected rabbits and the other two displayed no obvious lesion. No obvious injury was seen in rabbits in other groups (Supplementary Figure 6C).

## Discussion

The mechanisms underlying the presence of HEV in the male reproductive system, especially the persistent shedding of HEV in the ejaculates of immunocompromised chronic hepatitis E patients, are still unclear. Moreover, the impact of HEV replication on the male reproductive system remains poorly understood. In this study, using an ex vivo model of human testis and primary human Sertoli cells, we provide here the ﬁrst evidence that HEV can infect human testis and the testis is prone to HEV3 infection rather than HEV1, resulting in the generation of infectious progeny virions and tissue injury. Furthermore, tacrolimus treatment increased the susceptibility and replication efficiency of HEV in both the testis and Sertoli cells.

Previous studies demonstrated HEV3 viral particles in the ejaculate of chronically HEV-infected men [24, 25]. Interestingly, HEV could be detected in much higher concentrations and longer duration in the ejaculate in comparison to the blood or stool. Our results provide an explanation for this clinical manifestation that HEV3 can indeed infect and replicate within human testis explants. Notably, the virus strains we used here are all wild-type HEVs isolated from stool samples of hepatitis E patients or infected animals, and wild-type strains are known to be extremely difficult to culture in cells. Previous studies frequently used the Kernow-C1 p6 cloned virus for in vitro replication/infection [10, 14]. It seems that this strain has a more favorable in vitro growth efficiency in hepatic cells or extrahepatic cells than wild-type HEVs. However, existing evidence suggests that the viral characteristics may be changed with this strain because of its cell-adapted nature [29]. The use of wild-type HEV3 demonstrates the susceptibility of human testis explants to HEV. We observed the release of infectious HEV3 virions into the supernatants from the inoculated testis explants, which was further validated by the successful infection of a Mongolian gerbil using these supernatants. This result demonstrates that testicular cells support the thorough life cycle of HEV3. Specifically, our study uncovered that Sertoli cells can be infected with HEV. To infect these cells, HEV must cross the blood-testis barrier formed by Sertoli cell tight junctions. Direct infection of Sertoli cells is supported by our results in primary human cells. These data demonstrate that HEV replicates in germ cells and suggest that the virus might be able to bypass the blood-testis barrier by infecting Sertoli cells.

HEV3 slightly induced tissue injury of the testis explants and affected the tissue morphology. It is essential to note that the long-term impact on the reproductive health of male solid organ transplant recipients, who constantly taking tacrolimus for immunosuppression (more than years), is a subject of debate and warrants further study [30]. However, the secretome of the testis explants showed no obvious alteration. This observation may be attributed to the immunosuppressive effects of tacrolimus, which could dampen the immune response. Notably, HEV alters the secretion profile of primary human Sertoli cells. The levels of several pro-inflammatory cytokines and chemokines significantly increased in the supernatants of the HEV-infected Sertoli cells. Such a pro-inflammatory microenvironment may lead to the destruction of the blood-testis barrier. Indeed, a previous animal study reported that the level of zona occludin-1 in HEV RNA-positive testicular tissues was decreased significantly compared to mock-infected Mongolian gerbils [31]. Our observation suggests that the possible association between HEV infection and testicular injury should be closely monitored in patients with chronic HEV infection.

Our findings highlight a distinct susceptibility between HEV genotypes to the human testis and Sertoli cells. HEV3 but not HEV1 can readily infect testis explants and primary human Sertoli cells. We also tested other HEV genotypes including HEV4 and HEV-C1 (rat HEV). None of these genotypes showed obvious signs of infection in the study models. Indeed, HEV-associated extrahepatic manifestations are more frequently reported in patients with HEV3 infection [5]. To date, detection of HEV RNA in human semen samples has only been comprehensively confirmed in patients with chronic HEV3 infections [24]. HEV4 has been detected in infertile male semen samples in a study conducted in Kunming, China [15]. However, further investigation by our group detected no HEV RNA in infertile male samples collected in Beijing, China, indicating possible differences in the prevalence of different regions [16]. Similar studies conducted in Egypt [18] and Germany [17], where HEV1 and HEV3 are prevalent, respectively, also detect no HEV RNA in infertile male samples. In natural settings, studies have found HEV4 in semen samples of pigs in China [32] and HEV3 in testis tissues of wild boars in Spain [33]. Moreover, a recent study also detected HEV-C1 RNA in wild rats’ testis tissues [34]. Whether the HEV tropism for the male reproductive tract is restricted to certain HEV genotypes warrants further investigation.

The previous study could not find any detectable HEV RNA in the ejaculate of six immunocompetent individuals with acute HEV infection. In addition, HEV RNA was undetectable in the testis of 12 experimentally infected immunocompetent pigs [24]. This indicates that testicular HEV replication may primarily occur in immunocompromised patients. In our ex vivo model, human testis explants treated with tacrolimus, a commonly used immunosuppressant, presented with increased efficiency of HEV replication. The susceptibility of tacrolimus-treated testis explants also increased. This phenomenon was observed as well in the experiment of HEV infection of primary human Sertoli cells. Moreover, we found that HEV1 could infect Sertoli cells only when tacrolimus was added. These results provide plausible explanations for the observations that no HEV was detected in acute hepatitis E patients. Previous studies demonstrated that clinical tacrolimus therapy is the main predictive factor for hepatitis E chronicity in solid organ transplant recipients [35] and tacrolimus facilitates HEV replication in vitro [36]. In our study, tacrolimus increased the replication efficiency of both HEV1 and HEV3 in testicular cells. The results suggest that the level of immunosuppression is an essential factor associated with the presence of HEV in the male reproductive system. The persistence of HEV infection in a host is also important for the extrahepatic replication of HEV. A case report found that a nonimmunocompromised patient developed chronic hepatitis E and manifested with cutaneous CD30^+^ T cell lymphoproliferative disorder [37]. HEV replication was found in the dermal endothelium of this patient. In animal experiments, HEV extrahepatic replication was also observed in immunocompetent nonhuman primates [38] and rabbits [9] presented with persistent HEV infection. Taken together, the presence of HEV in the ejaculates should be monitored in patients with chronic hepatitis E of all HEV genotypes.

The animal model is a vital tool for studying viral-host interplay for chronic hepatitis E. We have previously established an immunocompromised rabbit model for the study of chronic HEV infection [11]. In the present study, we employed this model to study the HEV-host interaction in the testis. At the chronic phase (13 wpi) of HEV3 (rabbit HEV) infection, HEV RNA can be detected in the tacrolimus-treated rabbits’ testis tissues. Transcriptome analysis of testis tissues in HEV-infected versus mock-infected rabbits showed that many DEGs related to spermatogenesis were down-regulated. This result indicated that at least in our model, chronic HEV infection posed a negative influence on male reproductive health. Of note, previous animal studies using immunocompetent mice [39] and gerbils [31] of acute HEV4 infection also found that HEV can replicate in the male reproductive systems and induce injury in the testis. The real influence of HEV replication on male reproductive health should be thoroughly investigated in humans in the future. Our findings advance the application of the immunocompromised rabbit model by investigation into the male reproductive system and extrahepatic tissue transcriptome under the context of chronic HEV infection.

In conclusion, we demonstrated that HEV replicates in the human testis ex vivo and infects Sertoli cells. The replication efficiency of HEV could be augmented by tacrolimus and might be related to viral persistence in the ejaculate of patients with chronic hepatitis E. Our findings also reveal that HEV might have a mild-to-moderate deleterious effect on the morphology of the human testis in culture and induced abnormal pro-inflammatory cytokine and chemokine secretion in primary human Sertoli cells. These results doesn't necessarily indicate any association between HEV infection and male infertility. In rabbits with chronic HEV infection, HEV exerted a negative influence on spermatogenesis in testis tissues. Overall, these results underscore the need for further investigation of the impact of HEV on male reproductive health especially in patients with chronic hepatitis E. The ex vivo model of HEV infection of the human testis we developed provides a valuable tool for the testing of therapeutic strategies and advancing our understanding of HEV infection dynamics in the male reproductive system.

## Supplementary Material

Supplementary_Materials_Testis_EMI_revised_0208-clean

Supplementary_Table_S1

Supplementary_figures
