# Supplementary material for: Hepatitis E virus infects human testicular tissue and Sertoli cells
Source: Emerg Microbes Infect. 2024 Mar 22;13(1):2332657. doi: 10.1080/22221751.2024.2332657 (PMC11057402; doi:10.1080/22221751.2024.2332657)
Supplement: Supplementary_Materials_Testis_EMI_revised_0208-clean [file TEMI_A_2332657_SM5727.docx]

**Supplementary Materials**

**Animals experiments**

A female and three male Mongolian gerbils (13-week-old) were purchased from Sipeifu Biotech, Beijing, China. Serum and fecal samples were collected weekly and tested for HEV RNA and/or anti-HEV antibodies before enrollment to exclude current or previous HEV infection. Gerbil were housed in a cage with adequate water and food.

Three-month-old Japanese white rabbits weighing about 2.5-3.0 kg were randomly selected. Serum and fecal samples were collected and tested for HEV RNA and/or anti-HEV antibodies before enrollment. Rabbits were housed in separate cages with adequate water and food. Tacrolimus was administrated daily via gavage for seven consecutive days with 1 mg/d once daily after HEV inoculation and reduced to every two days from the second week. The animal experiments were approved by the Committee of Laboratory Animal Welfare and Ethics, Peking University Health Science Center and the Committee on the Ethics of Animal Experiments of the Harbin Veterinary Research Institute, Chinese Academy of Agricultural Sciences.

**Immunofluorescence staining**

Human testis tissues were fixed in testicular tissue fixation buffer (G1121, Servicebio, Wuhan, China) for 12-16 hours, and then embedded in paraffin. The paraffin sections were heated at 95 °C in EDTA antigen repair buffer (ZLI-9071, ZSGB-BIO; Beijing, China) for 30 minutes and cooled into room temperature. The sections were then blocked in 10% donkey serum (in 0.1 M PBS) with 0.3% Triton X-100 for 1 hour at room temperature, and incubated with the primary antibodies in 1% donkey serum (in 0.1 M PBS) at 4 °C overnight which includes rabbit monoclonal anti-PCNA (1:100; 13110S, Cell Signaling Technology, USA), rabbit polyclonal anti-ORF2 (1:100; bs-16495R, Bioss, Woburn, USA); mouse monoclonal anti-DDX4 (1:100; ab27591, Abcam, Cambridge, UK); mouse monoclonal anti-SOX9 (1:100; H00006662-M01, Abnova) and mouse monoclonal anti-alpha smooth muscle Actin (1:100; ab7817, Abcam), respectively. After wash in PBS for three times, tissues were incubated with the following secondary antibodies at room temperature for 1 hour: Cy3-conjugated AffiniPure donkey polyclonal anti-mouse IgG (H+L) and Alexa Fluor 488-conjugated AffiniPure donkey polyclonal anti-rabbit IgG (H+L) (both 1:500; Jackson ImmunoResearch Laboratories, Philadelphia, PA, USA). The tissues were counterstained with the nuclear marker DAPI (100 ng/mL, Beyotime, Jiangsu, China) for 10 minutes at room temperature. After three washes in PBS, the slides were mounted in Gel-Mount medium and observed under a confocal microscope (Zeiss LSM880, Carl Zeiss Microscopy GmbH, Jena, Germany) at excitation wavelengths of 488 nm (green), 555 nm (red) and 405 nm (blue).

**Isolation and culture of primary human Sertoli cells**

Primary human Sertoli cells were purchased from Zhejiang Meisen Cell Technology Co., Ltd, and were cultured in a primary cell culture medium (purchased from Zhejiang Meisen Cell Technology Co., Ltd, Hangzhou, China). When primary Sertoli cell reached to a confluency of 80%, the cells were incubated with approximately 1×10^7^ copies HEV at 37℃, 5% CO_2_. After 24 hours, cell supernatants were removed by washing with 1×PBS, then fresh primary cell culture medium were added. Cell supernatants were collected and stored in -80℃ for downstream experiments.

**Transcriptome profiling**

Whole-genome gene expression analysis was performed in testis tissue of rabbit from Mock, Mock+Tac and HEV-3ra group. Total RNA was extracted from the cryopreserved testes by using TRIzol Reagent (Invitrogen, CA, USA). After the total RNA samples were tested to be qualified, the MGIEasy RNA library preparation kit was used for library construction. After purification, the double stranded PCR library was unzipped and then looped to form single stranded circular DNA. The high-throughput sequencing was performed using the MGI DNBSEQ-T7 sequencing platform with a sequencing read length of PE150. Principal component analysis (PCA) was calculated. Differential expression analysis was performed using the DESeq2 package to identify genes that were expressed differently between two groups.

**Luminex liquid suspension chip detection**

The levels of cytokines (CTACK, Eotaxin, Basic FGF, GCSF, GMCSF, GROα, HGF, IFNα2, IFNγ, IL1α, IL1β, IL1ra, IL2, IL-2Rα, IL3, IL4, IL-5, IL6, IL7, IL8, IL9, IL10, IL12(P40), IL12 (P70), IL13, IL15, IL16, IL17, IL18, IP10, LIF, MCP1, MCP3, MCSF, MIF, MIG, MIP1α, MIP1β, β-NGF, PDGFBB, RANTES, SCF1α, SCFGβ, SDF1α, TNFα, TNFβ, TRAIL, VEGF) were detected by Wayen Biotechnology (Shanghai, China) using the Human 48-Plex Luminex assay. The Bio-Plex Pro Human Cytokine Screening 48-plex panel was used following the manufacturer's instructions. In brief, supernatants (50 µl) from the primary human Sertoli cells and human testicular tissues were added to each well of a 96 well plate, and then incubated with detection antibody for 30 min. Then 50 μL diluted Streptavidin-PE were added to each well and incubated for 10 min. Bio-Plex 200 system (Luminex Corporation, Austin, TX, USA) was used for data acquisition.

**Statistical analysis**

Prism 9 software (Graphic software) was used for charts and statistical analyses. wilcoxon signed-rank test was used for statistical analysis to determine significant differences. Asterisks denote statistical significance (∗*p* < 0.05; ∗∗*p* < 0.01; ∗∗∗ *p* < 0.001). The data are reported as the mean ± SEM. The exact value of n representing number of animals are included in each figure legend.
