# Supplementary figures and images for: Hepatitis E virus infects human testicular tissue and Sertoli cells
Source: Emerg Microbes Infect. 2024 Mar 22;13(1):2332657. doi: 10.1080/22221751.2024.2332657 (PMC11057402; doi:10.1080/22221751.2024.2332657)

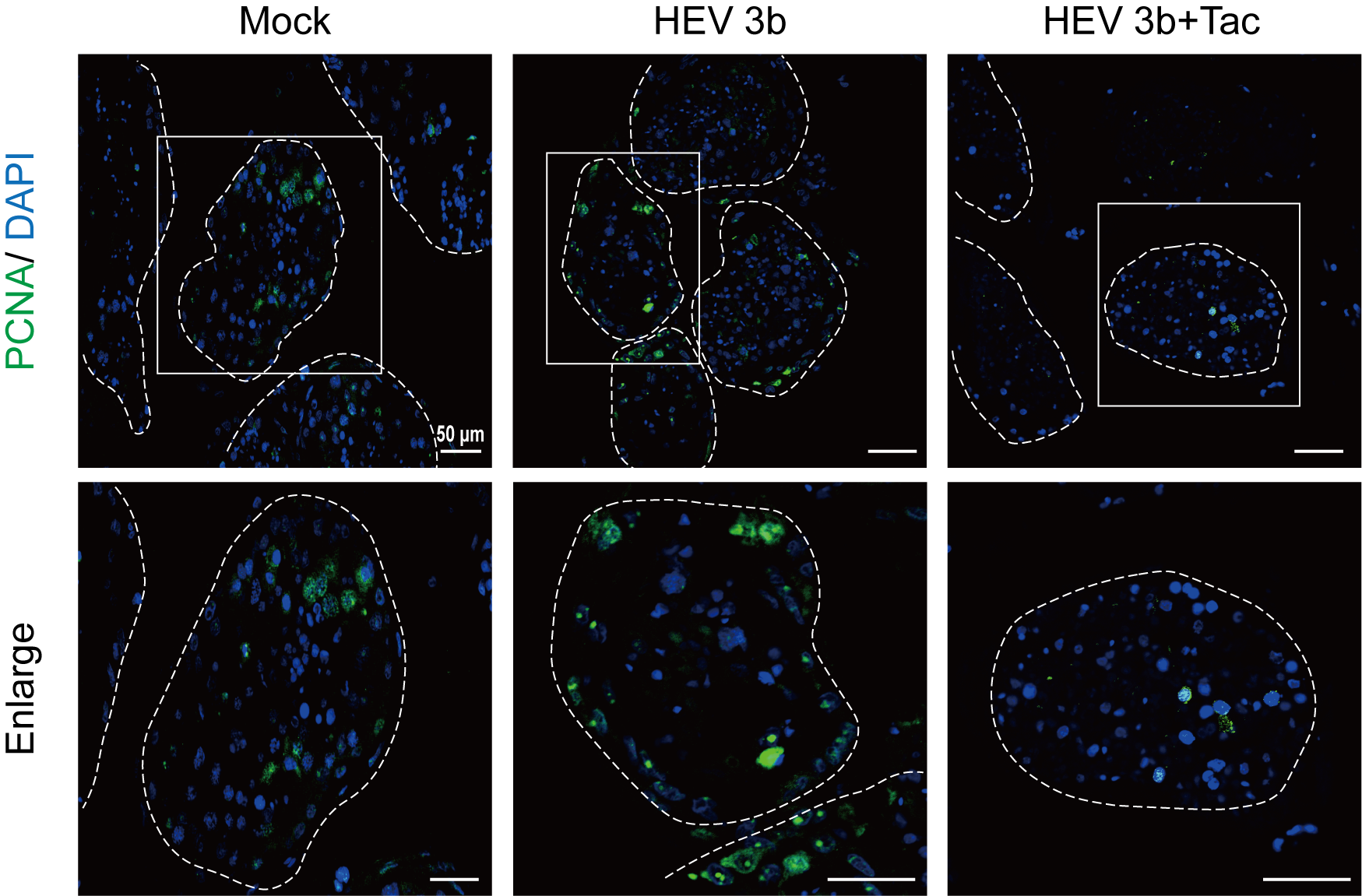

Supplement: Supplementary_figures [file TEMI_A_2332657_SM5725.zip › Fig S1.tif]

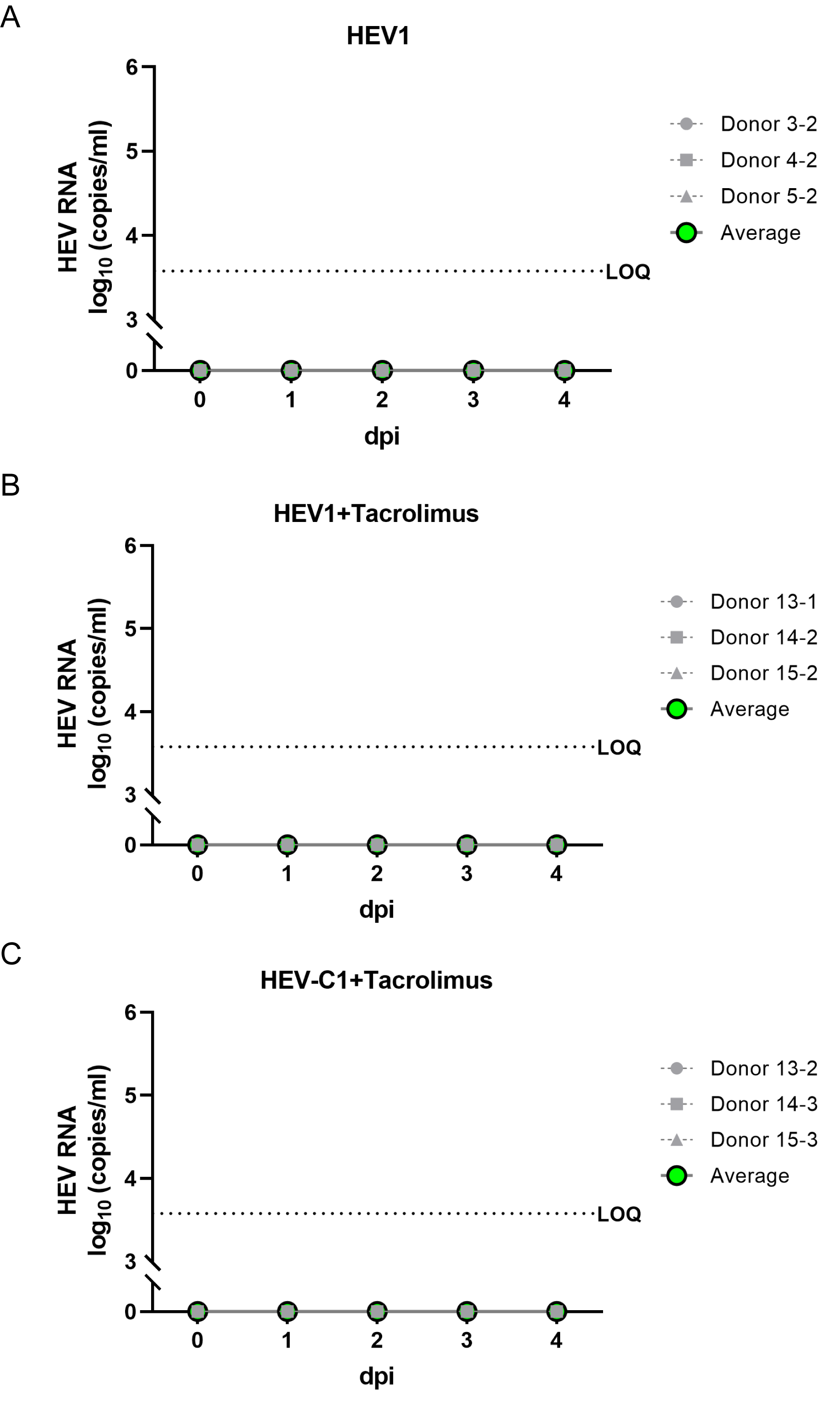

Supplement: Supplementary_figures [file TEMI_A_2332657_SM5725.zip › Fig S2.tif]

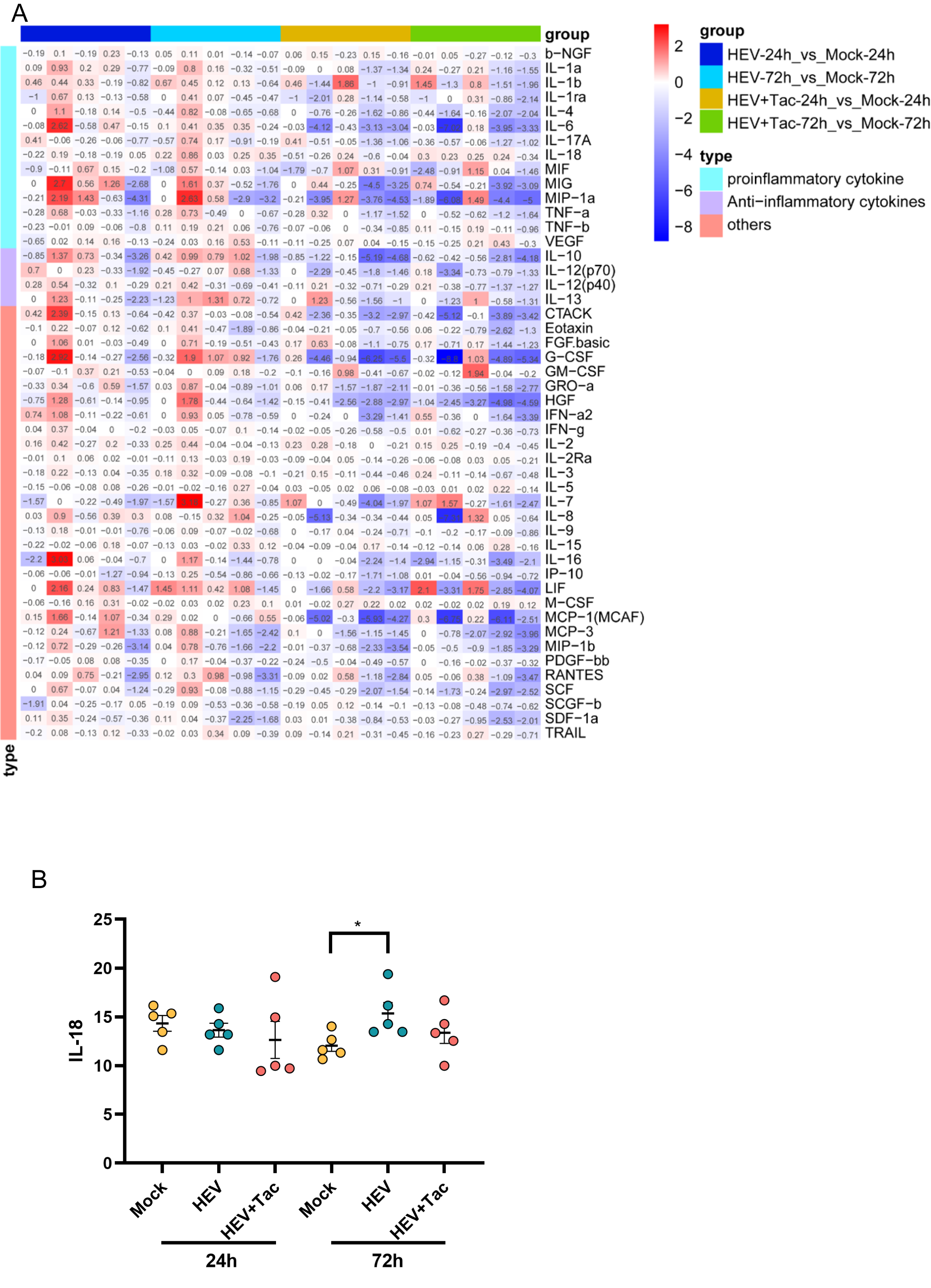

Supplement: Supplementary_figures [file TEMI_A_2332657_SM5725.zip › Fig S3.tif]

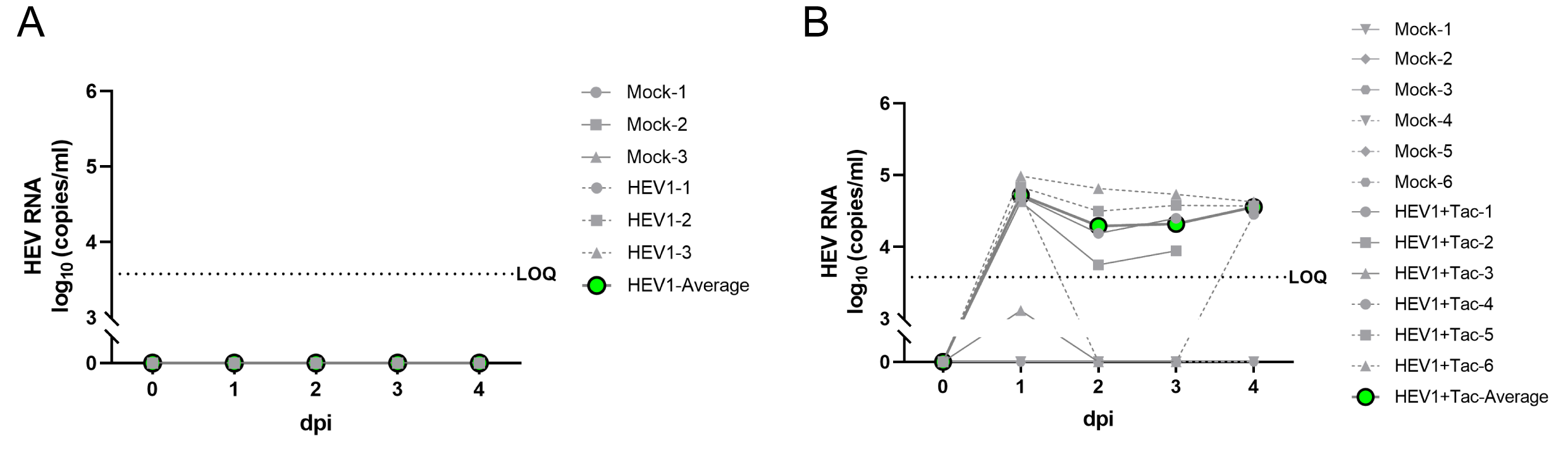

Supplement: Supplementary_figures [file TEMI_A_2332657_SM5725.zip › Fig S4.tif]

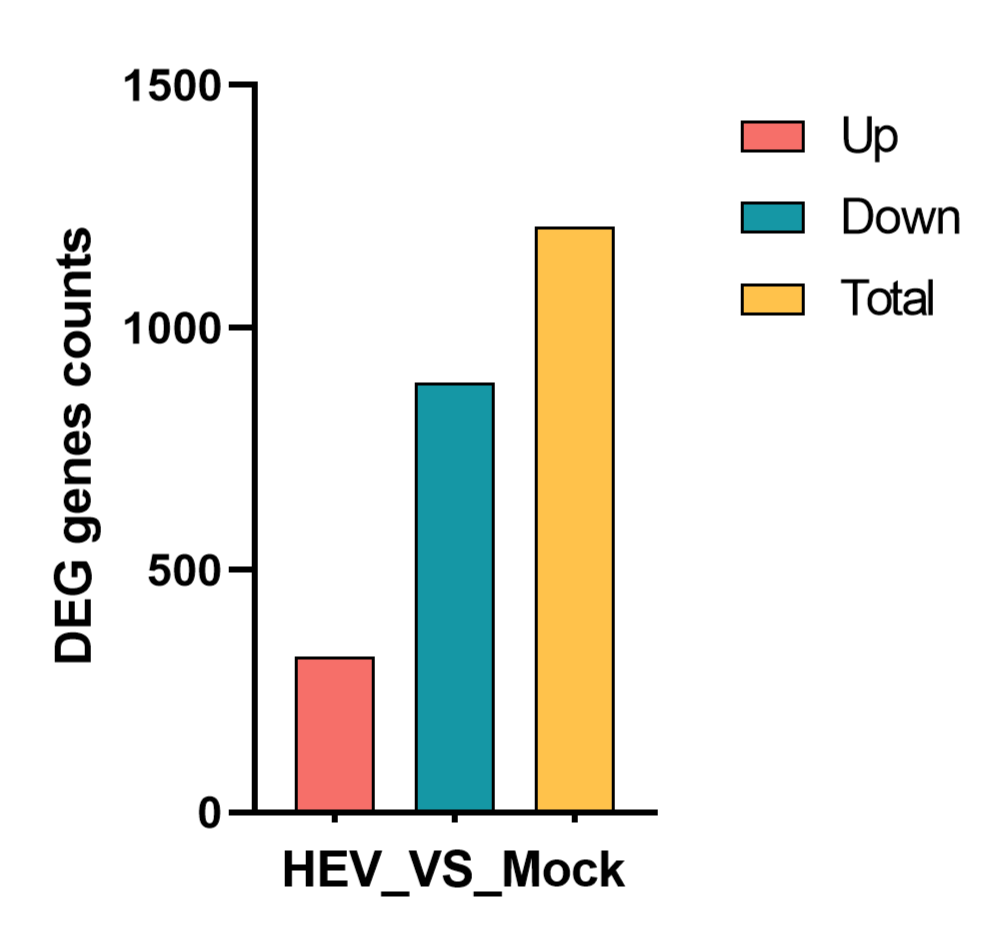

Supplement: Supplementary_figures [file TEMI_A_2332657_SM5725.zip › Fig S5.tif]

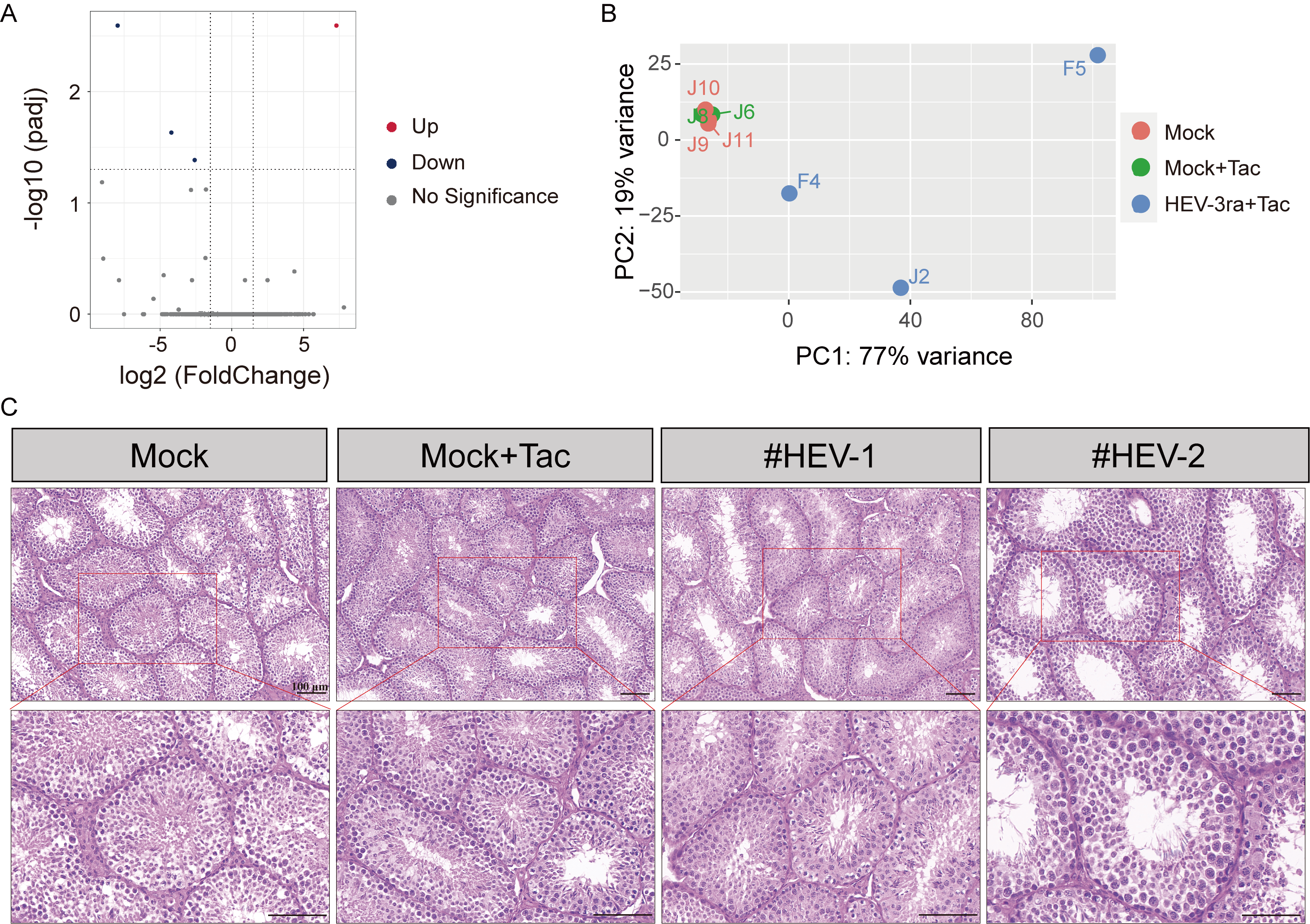

Supplement: Supplementary_figures [file TEMI_A_2332657_SM5725.zip › Fig S6.tif]
